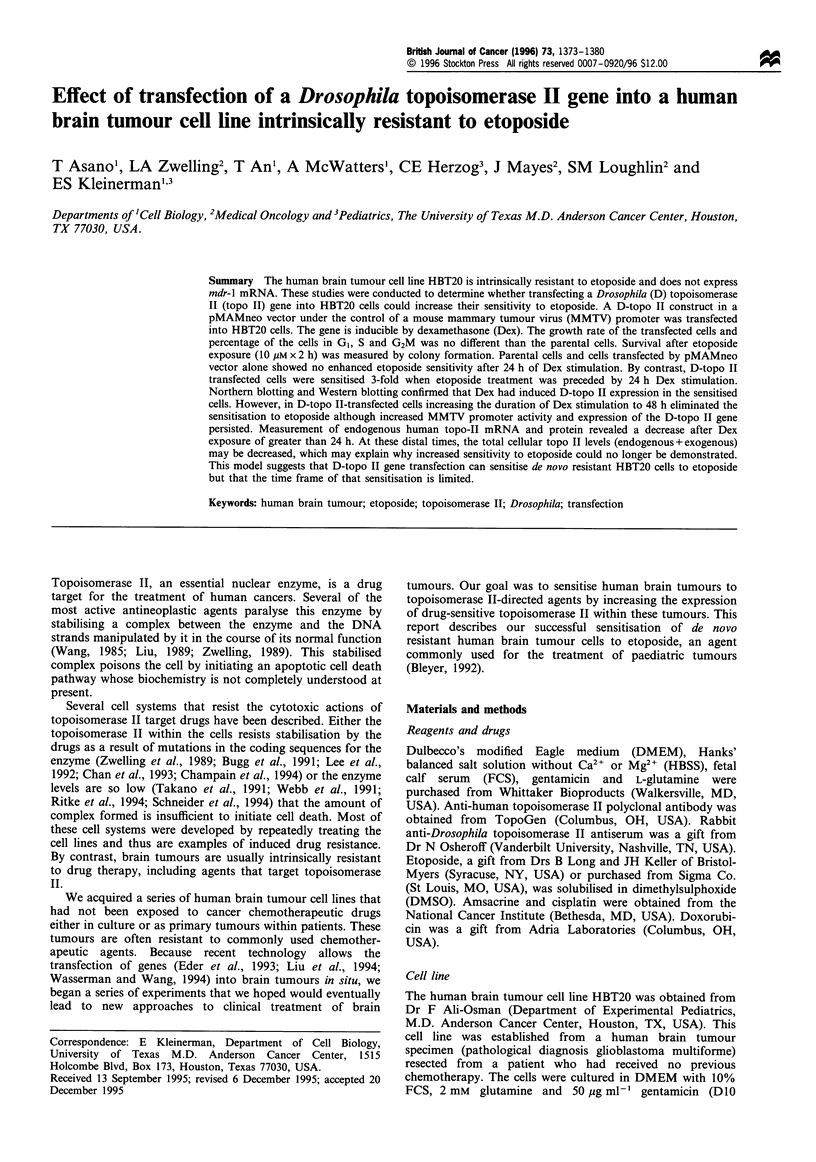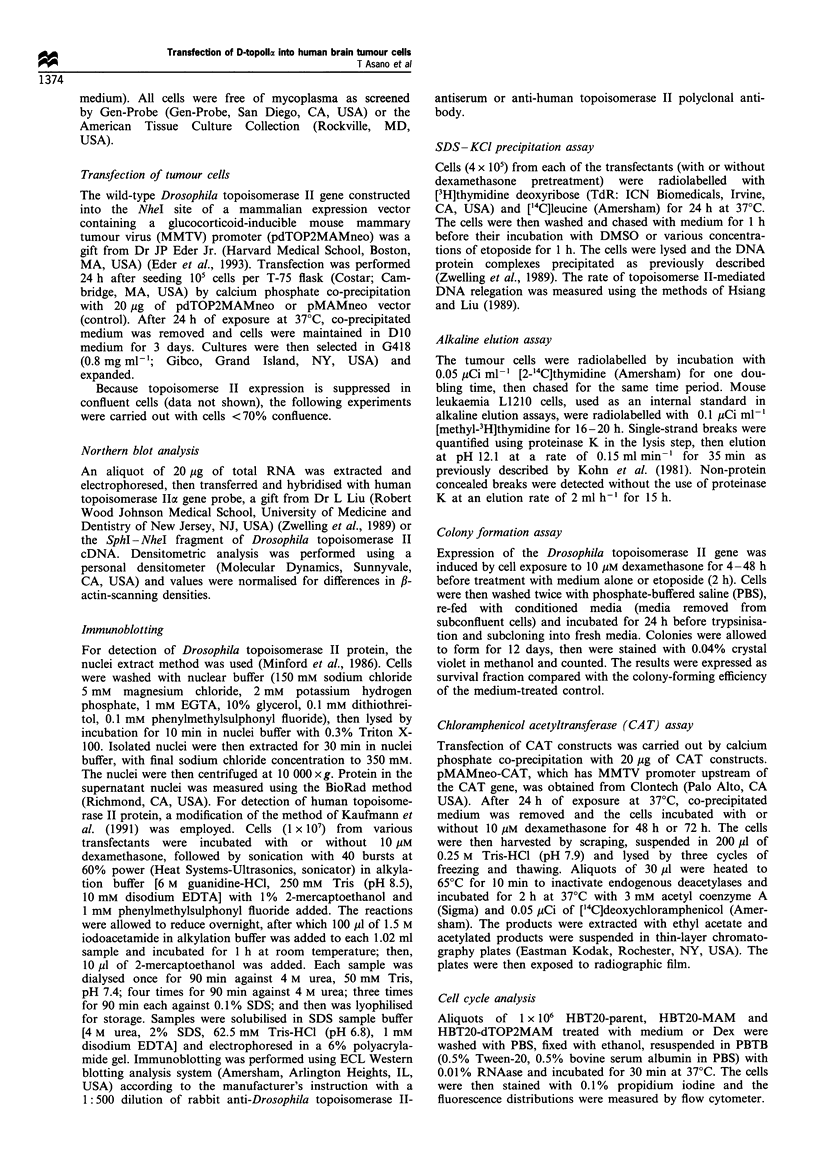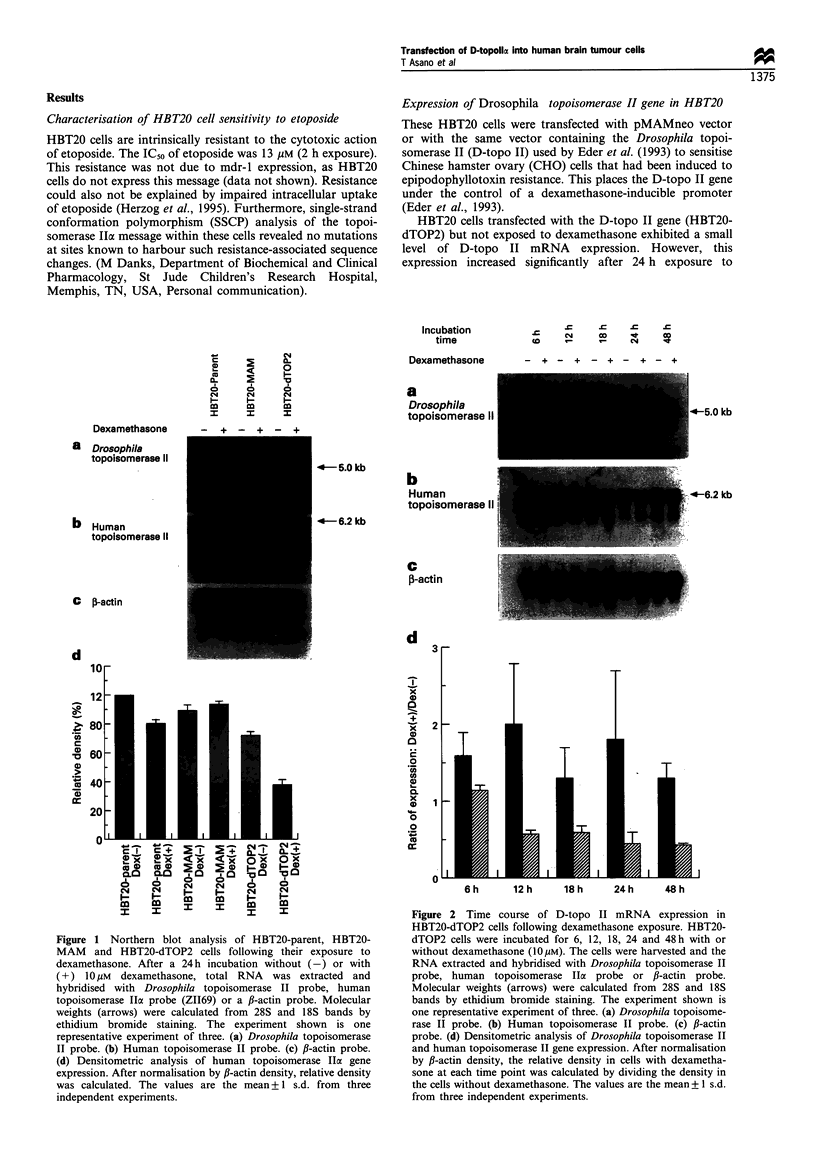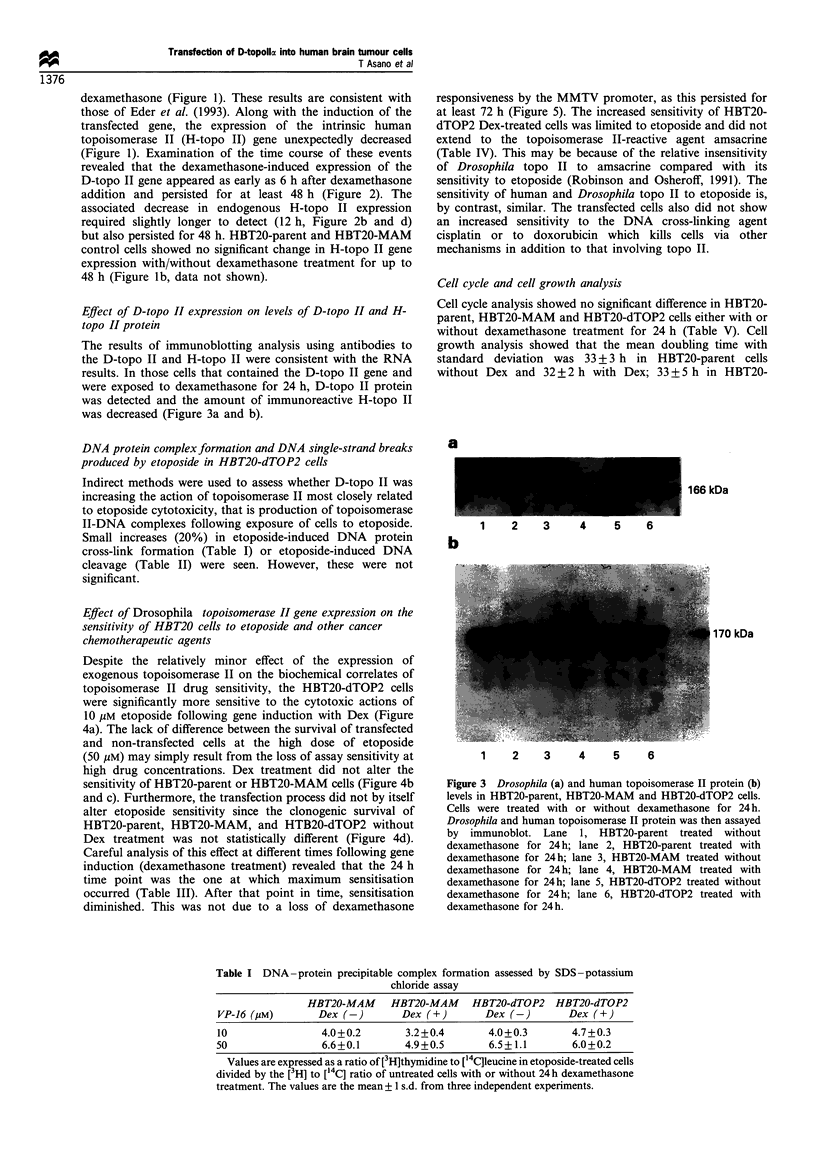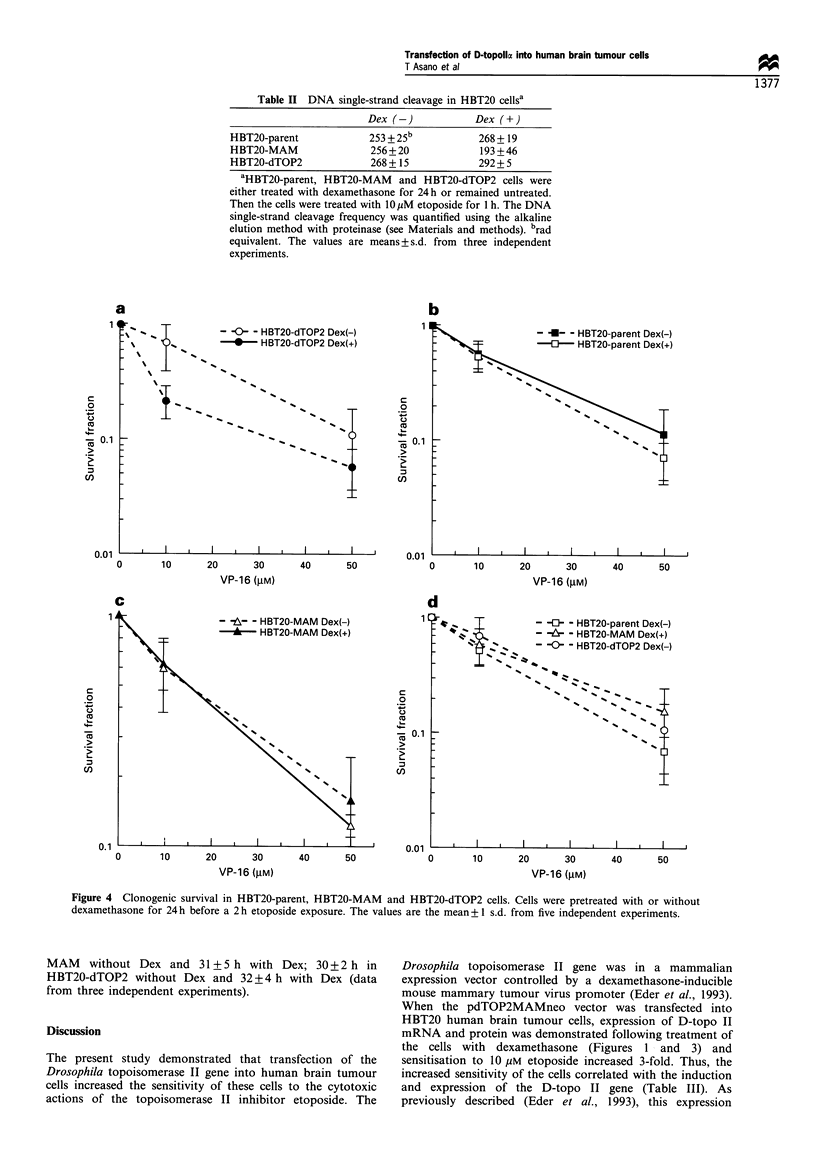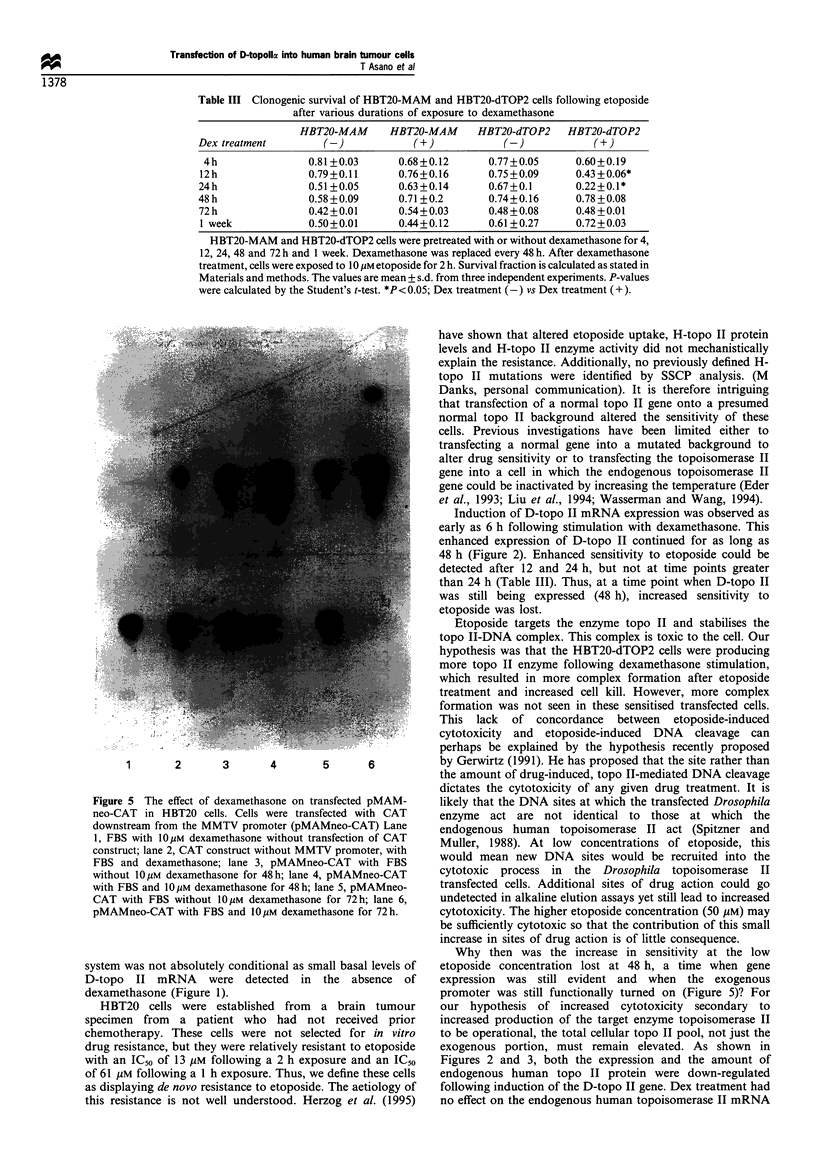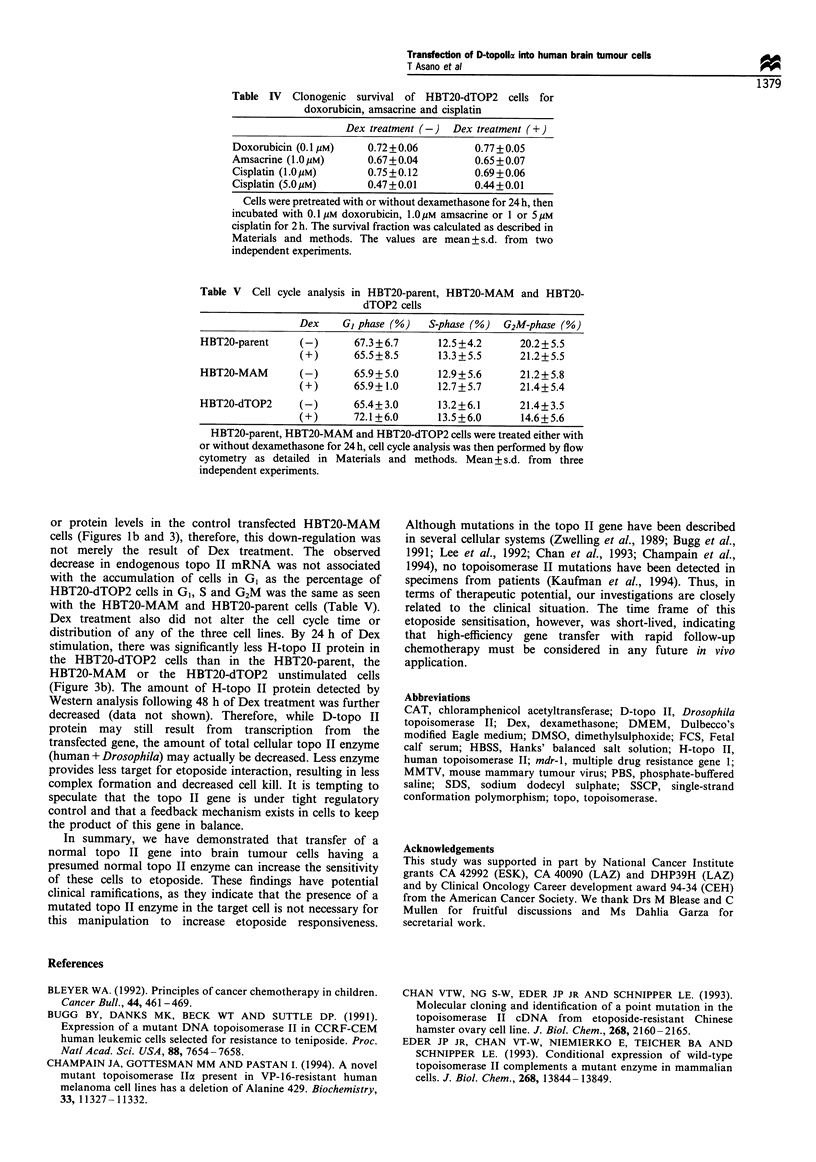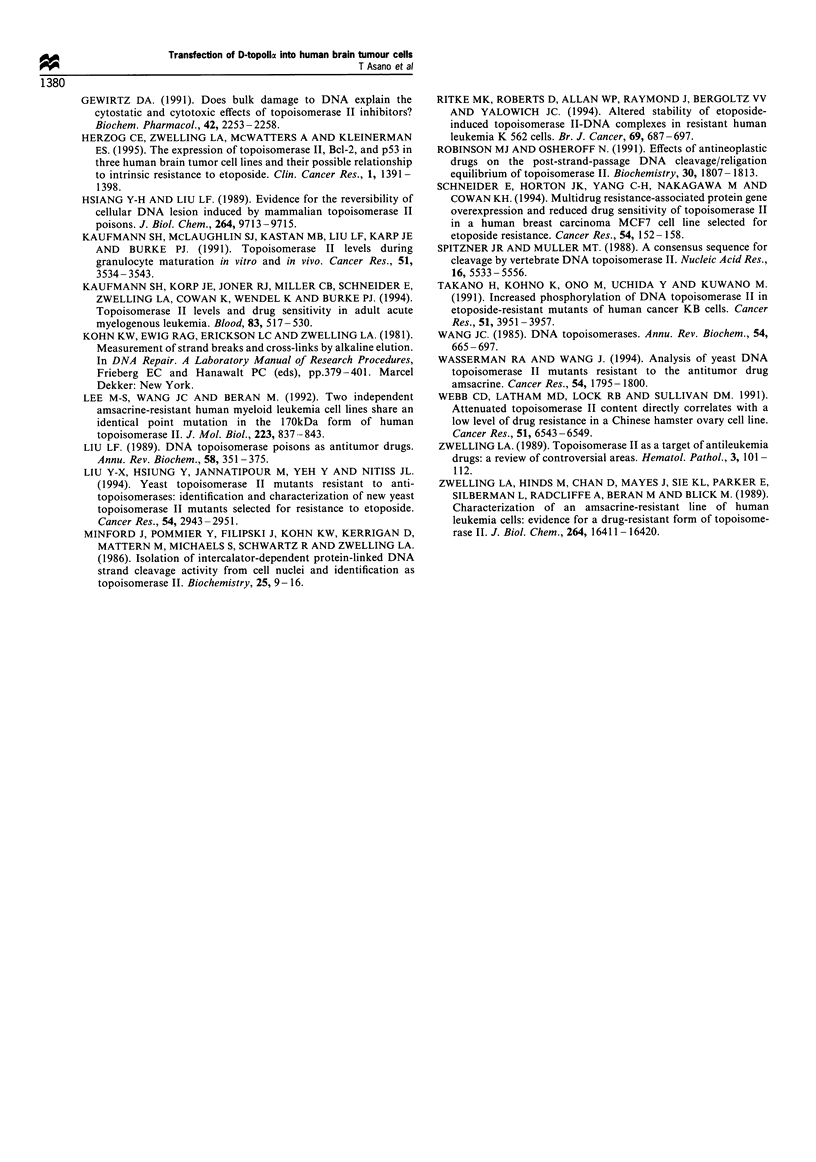# Effect of transfection of a Drosophila topoisomerase II gene into a human brain tumour cell line intrinsically resistant to etoposide.

**DOI:** 10.1038/bjc.1996.261

**Published:** 1996-06

**Authors:** T. Asano, L. A. Zwelling, T. An, A. McWatters, C. E. Herzog, J. Mayes, S. M. Loughlin, E. S. Kleinerman

**Affiliations:** Department of Cell Biology, University of Texas M.D. Anderson Cancer Center, Houston 77030, USA.

## Abstract

**Images:**


British Journal of Cancer (1996) 73, 1373-1380
© 1996 Stockton Press All rights reserved 0007-0920/96 $12.00

### Effect of transfection of a *Drosophila* topoisomerase II gene into a human brain tumour cell line intrinsically resistant to etoposide

T Asano[1], LA Zwelling[2], T An[1], A McWatters[1], CE Herzog[3], J Mayes[2], SM Loughlin[2] and ES Kleinerman[1,3]

*Departments of [1]Cell Biology, [2]Medical Oncology and [3]Pediatrics, The University of Texas M.D. Anderson Cancer Center, Houston, TX 77030, USA.*

**Summary** The human brain tumour cell line HBT20 is intrinsically resistant to etoposide and does not express *mdr*-1 mRNA. These studies were conducted to determine whether transfecting a *Drosophila* (D) topoisomerase II (topo II) gene into HBT20 cells could increase their sensitivity to etoposide. A D-topo II construct in a pMAMneo vector under the control of a mouse mammary tumour virus (MMTV) promoter was transfected into HBT20 cells. The gene is inducible by dexamethasone (Dex). The growth rate of the transfected cells and percentage of the cells in $G_1$, S and $G_2M$ was no different than the parental cells. Survival after etoposide exposure (10 $\mu$M × 2 h) was measured by colony formation. Parental cells and cells transfected by pMAMneo vector alone showed no enhanced etoposide sensitivity after 24 h of Dex stimulation. By contrast, D-topo II transfected cells were sensitised 3-fold when etoposide treatment was preceded by 24 h Dex stimulation. Northern blotting and Western blotting confirmed that Dex had induced D-topo II expression in the sensitised cells. However, in D-topo II-transfected cells increasing the duration of Dex stimulation to 48 h eliminated the sensitisation to etoposide although increased MMTV promoter activity and expression of the D-topo II gene persisted. Measurement of endogenous human topo-II mRNA and protein revealed a decrease after Dex exposure of greater than 24 h. At these distal times, the total cellular topo II levels (endogenous + exogenous) may be decreased, which may explain why increased sensitivity to etoposide could no longer be demonstrated. This model suggests that D-topo II gene transfection can sensitise *de novo* resistant HBT20 cells to etoposide but that the time frame of that sensitisation is limited.

**Keywords:** human brain tumour; etoposide; topoisomerase II; *Drosophila*; transfection

Topoisomerase II, an essential nuclear enzyme, is a drug target for the treatment of human cancers. Several of the most active antineoplastic agents paralyse this enzyme by stabilising a complex between the enzyme and the DNA strands manipulated by it in the course of its normal function (Wang, 1985; Liu, 1989; Zwelling, 1989). This stabilised complex poisons the cell by initiating an apoptotic cell death pathway whose biochemistry is not completely understood at present.

Several cell systems that resist the cytotoxic actions of topoisomerase II target drugs have been described. Either the topoisomerase II within the cells resists stabilisation by the drugs as a result of mutations in the coding sequences for the enzyme (Zwelling *et al.*, 1989; Bugg *et al.*, 1991; Lee *et al.*, 1992; Chan *et al.*, 1993; Champain *et al.*, 1994) or the enzyme levels are so low (Takano *et al.*, 1991; Webb *et al.*, 1991; Ritke *et al.*, 1994; Schneider *et al.*, 1994) that the amount of complex formed is insufficient to initiate cell death. Most of these cell systems were developed by repeatedly treating the cell lines and thus are examples of induced drug resistance. By contrast, brain tumours are usually intrinsically resistant to drug therapy, including agents that target topoisomerase II.

We acquired a series of human brain tumour cell lines that had not been exposed to cancer chemotherapeutic drugs either in culture or as primary tumours within patients. These tumours are often resistant to commonly used chemotherapeutic agents. Because recent technology allows the transfection of genes (Eder *et al.*, 1993; Liu *et al.*, 1994; Wasserman and Wang, 1994) into brain tumours *in situ*, we began a series of experiments that we hoped would eventually lead to new approaches to clinical treatment of brain tumours. Our goal was to sensitise human brain tumours to topoisomerase II-directed agents by increasing the expression of drug-sensitive topoisomerase II within these tumours. This report describes our successful sensitisation of *de novo* resistant human brain tumour cells to etoposide, an agent commonly used for the treatment of paediatric tumours (Bleyer, 1992).

Correspondence: E Kleinerman, Department of Cell Biology, University of Texas M.D. Anderson Cancer Center, 1515 Holcombe Blvd, Box 173, Houston, Texas 77030, USA.
Received 13 September 1995; revised 6 December 1995; accepted 20 December 1995

### Materials and methods

### Reagents and drugs

Dulbecco's modified Eagle medium (DMEM), Hanks' balanced salt solution without $Ca^{2+}$ or $Mg^{2+}$ (HBSS), fetal calf serum (FCS), gentamicin and L-glutamine were purchased from Whittaker Bioproducts (Walkersville, MD, USA). Anti-human topoisomerase II polyclonal antibody was obtained from TopoGen (Columbus, OH, USA). Rabbit anti-*Drosophila* topoisomerase II antiserum was a gift from Dr N Osheroff (Vanderbilt University, Nashville, TN, USA). Etoposide, a gift from Drs B Long and JH Keller of Bristol-Myers (Syracuse, NY, USA) or purchased from Sigma Co. (St Louis, MO, USA), was solubilised in dimethylsulphoxide (DMSO). Amsacrine and cisplatin were obtained from the National Cancer Institute (Bethesda, MD, USA). Doxorubicin was a gift from Adria Laboratories (Columbus, OH, USA).

### Cell line

The human brain tumour cell line HBT20 was obtained from Dr F Ali-Osman (Department of Experimental Pediatrics, M.D. Anderson Cancer Center, Houston, TX, USA). This cell line was established from a human brain tumour specimen (pathological diagnosis glioblastoma multiforme) resected from a patient who had received no previous chemotherapy. The cells were cultured in DMEM with 10% FCS, 2 mM glutamine and 50 $\mu$g ml$^{-1}$ gentamicin (D10

medium). All cells were free of mycoplasma as screened by Gen-Probe (Gen-Probe, San Diego, CA, USA) or the American Tissue Culture Collection (Rockville, MD, USA).

*Transfection of tumour cells*

The wild-type *Drosophila* topoisomerase II gene constructed into the *Nhe*I site of a mammalian expression vector containing a glucocorticoid-inducible mouse mammary tumour virus (MMTV) promoter (pdTOP2MAMneo) was a gift from Dr JP Eder Jr. (Harvard Medical School, Boston, MA, USA) (Eder *et al.*, 1993). Transfection was performed 24 h after seeding $10^5$ cells per T-75 flask (Costar; Cambridge, MA, USA) by calcium phosphate co-precipitation with 20 μg of pdTOP2MAMneo or pMAMneo vector (control). After 24 h of exposure at 37°C, co-precipitated medium was removed and cells were maintained in D10 medium for 3 days. Cultures were then selected in G418 (0.8 mg $ml^{-1}$; Gibco, Grand Island, NY, USA) and expanded.

Because topoisomerse II expression is suppressed in confluent cells (data not shown), the following experiments were carried out with cells <70% confluence.

*Northern blot analysis*

An aliquot of 20 μg of total RNA was extracted and electrophoresed, then transferred and hybridised with human topoisomerase IIα gene probe, a gift from Dr L Liu (Robert Wood Johnson Medical School, University of Medicine and Dentistry of New Jersey, NJ, USA) (Zwelling *et al.*, 1989) or the *Sph*I–*Nhe*I fragment of *Drosophila* topoisomerase II cDNA. Densitometric analysis was performed using a personal densitometer (Molecular Dynamics, Sunnyvale, CA, USA) and values were normalised for differences in β-actin-scanning densities.

*Immunoblotting*

For detection of *Drosophila* topoisomerase II protein, the nuclei extract method was used (Minford *et al.*, 1986). Cells were washed with nuclear buffer (150 mM sodium chloride 5 mM magnesium chloride, 2 mM potassium hydrogen phosphate, 1 mM EGTA, 10% glycerol, 0.1 mM dithiothreitol, 0.1 mM phenylmethylsulphonyl fluoride), then lysed by incubation for 10 min in nuclei buffer with 0.3% Triton X-100. Isolated nuclei were then extracted for 30 min in nuclei buffer, with final sodium chloride concentration to 350 mM. The nuclei were then centrifuged at 10 000 × *g*. Protein in the supernatant nuclei was measured using the BioRad method (Richmond, CA, USA). For detection of human topoisomerase II protein, a modification of the method of Kaufmann *et al.* (1991) was employed. Cells ($1 \times 10^7$) from various transfectants were incubated with or without 10 μM dexamethasone, followed by sonication with 40 bursts at 60% power (Heat Systems-Ultrasonics, sonicator) in alkylation buffer [6 M guanidine-HCl, 250 mM Tris (pH 8.5), 10 mM disodium EDTA] with 1% 2-mercaptoethanol and 1 mM phenylmethylsulphonyl fluoride added. The reactions were allowed to reduce overnight, after which 100 μl of 1.5 M iodoacetamide in alkylation buffer was added to each 1.02 ml sample and incubated for 1 h at room temperature; then, 10 μl of 2-mercaptoethanol was added. Each sample was dialysed once for 90 min against 4 M urea, 50 mM Tris, pH 7.4; four times for 90 min against 4 M urea; three times for 90 min each against 0.1% SDS; and then was lyophilised for storage. Samples were solubilised in SDS sample buffer [4 M urea, 2% SDS, 62.5 mM Tris-HCl (pH 6.8), 1 mM disodium EDTA] and electrophoresed in a 6% polyacrylamide gel. Immunoblotting was performed using ECL Western blotting analysis system (Amersham, Arlington Heights, IL, USA) according to the manufacturer's instruction with a 1:500 dilution of rabbit anti-*Drosophila* topoisomerase II-antiserum or anti-human topoisomerase II polyclonal antibody.

*SDS–KCl precipitation assay*

Cells ($4 \times 10^5$) from each of the transfectants (with or without dexamethasone pretreatment) were radiolabelled with [$^3$H]thymidine deoxyribose (TdR: ICN Biomedicals, Irvine, CA, USA) and [$^{14}$C]leucine (Amersham) for 24 h at 37°C. The cells were then washed and chased with medium for 1 h before their incubation with DMSO or various concentrations of etoposide for 1 h. The cells were lysed and the DNA protein complexes precipitated as previously described (Zwelling *et al.*, 1989). The rate of topoisomerse II-mediated DNA relegation was measured using the methods of Hsiang and Liu (1989).

*Alkaline elution assay*

The tumour cells were radiolabelled by incubation with 0.05 μCi $ml^{-1}$ [2-$^{14}$C]thymidine (Amersham) for one doubling time, then chased for the same time period. Mouse leukaemia L1210 cells, used as an internal standard in alkaline elution assays, were radiolabelled with 0.1 μCi $ml^{-1}$ [methyl-$^3$H]thymidine for 16–20 h. Single-strand breaks were quantified using proteinase K in the lysis step, then elution at pH 12.1 at a rate of 0.15 ml $min^{-1}$ for 35 min as previously described by Kohn *et al.* (1981). Non-protein concealed breaks were detected without the use of proteinase K at an elution rate of 2 ml $h^{-1}$ for 15 h.

*Colony formation assay*

Expression of the *Drosophila* topoisomerase II gene was induced by cell exposure to 10 μM dexamethasone for 4–48 h before treatment with medium alone or etoposide (2 h). Cells were then washed twice with phosphate-buffered saline (PBS), re-fed with conditioned media (media removed from subconfluent cells) and incubated for 24 h before trypsinisation and subcloning into fresh media. Colonies were allowed to form for 12 days, then were stained with 0.04% crystal violet in methanol and counted. The results were expressed as survival fraction compared with the colony-forming efficiency of the medium-treated control.

*Chloramphenicol acetyltransferase (CAT) assay*

Transfection of CAT constructs was carried out by calcium phosphate co-precipitation with 20 μg of CAT constructs. pMAMneo-CAT, which has MMTV promoter upstream of the CAT gene, was obtained from Clontech (Palo Alto, CA USA). After 24 h of exposure at 37°C, co-precipitated medium was removed and the cells incubated with or without 10 μM dexamethasone for 48 h or 72 h. The cells were then harvested by scraping, suspended in 200 μl of 0.25 M Tris-HCl (pH 7.9) and lysed by three cycles of freezing and thawing. Aliquots of 30 μl were heated to 65°C for 10 min to inactivate endogenous deacetylases and incubated for 2 h at 37°C with 3 mM acetyl coenzyme A (Sigma) and 0.05 μCi of [$^{14}$C]deoxychloramphenicol (Amersham). The products were extracted with ethyl acetate and acetylated products were suspended in thin-layer chromatography plates (Eastman Kodak, Rochester, NY, USA). The plates were then exposed to radiographic film.

*Cell cycle analysis*

Aliquots of $1 \times 10^6$ HBT20-parent, HBT20-MAM and HBT20-dTOP2MAM treated with medium or Dex were washed with PBS, fixed with ethanol, resuspended in PBTB (0.5% Tween-20, 0.5% bovine serum albumin in PBS) with 0.01% RNAase and incubated for 30 min at 37°C. The cells were then stained with 0.1% propidium iodine and the fluorescence distributions were measured by flow cytometer.

### Results

### *Characterisation of HBT20 cell sensitivity to etoposide*

HBT20 cells are intrinsically resistant to the cytotoxic action of etoposide. The $IC_{50}$ of etoposide was 13 $\mu$M (2 h exposure). This resistance was not due to mdr-1 expression, as HBT20 cells do not express this message (data not shown). Resistance could also not be explained by impaired intracellular uptake of etoposide (Herzog *et al.*, 1995). Furthermore, single-strand conformation polymorphism (SSCP) analysis of the topoisomerase IIα message within these cells revealed no mutations at sites known to harbour such resistance-associated sequence changes. (M Danks, Department of Biochemical and Clinical Pharmacology, St Jude Children's Research Hospital, Memphis, TN, USA, Personal communication).

### *Expression of* Drosophila *topoisomerase II gene in HBT20*

These HBT20 cells were transfected with pMAMneo vector or with the same vector containing the *Drosophila* topoisomerase II (D-topo II) used by Eder *et al.* (1993) to sensitise Chinese hamster ovary (CHO) cells that had been induced to epipodophyllotoxin resistance. This places the D-topo II gene under the control of a dexamethasone-inducible promoter (Eder *et al.*, 1993).

HBT20 cells transfected with the D-topo II gene (HBT20-dTOP2) but not exposed to dexamethasone exhibited a small level of D-topo II mRNA expression. However, this expression increased significantly after 24 h exposure to

**Figure 1** Northern blot analysis of HBT20-parent, HBT20-MAM and HBT20-dTOP2 cells following their exposure to dexamethasone. After a 24 h incubation without (−) or with (+) 10 $\mu$M dexamethasone, total RNA was extracted and hybridised with *Drosophila* topoisomerase II probe, human topoisomerase IIα probe (ZII69) or a $\beta$-actin probe. Molecular weights (arrows) were calculated from 28S and 18S bands by ethidium bromide staining. The experiment shown is one representative experiment of three. (**a**) *Drosophila* topoisomerase II probe. (**b**) Human topoisomerase II probe. (**c**) $\beta$-actin probe. (**d**) Densitometric analysis of human topoisomerase IIα gene expression. After normalisation by $\beta$-actin density, relative density was calculated. The values are the mean ± 1 s.d. from three independent experiments.

**Figure 2** Time course of D-topo II mRNA expression in HBT20-dTOP2 cells following dexamethasone exposure. HBT20-dTOP2 cells were incubated for 6, 12, 18, 24 and 48 h with or without dexamethasone (10 $\mu$M). The cells were harvested and the RNA extracted and hybridised with *Drosophila* topoisomerase II probe, human topoisomerase IIα probe or $\beta$-actin probe. Molecular weights (arrows) were calculated from 28S and 18S bands by ethidium bromide staining. The experiment shown is one representative experiment of three. (**a**) *Drosophila* topoisomerase II probe. (**b**) Human topoisomerase II probe. (**c**) $\beta$-actin probe. (**d**) Densitometric analysis of *Drosophila* topoisomerase II and human topoisomerase II gene expression. After normalisation by $\beta$-actin density, the relative density in cells with dexamethasone at each time point was calculated by dividing the density in the cells without dexamethasone. The values are the mean ± 1 s.d. from three independent experiments.

dexamethasone (Figure 1). These results are consistent with those of Eder *et al.* (1993). Along with the induction of the transfected gene, the expression of the intrinsic human topoisomerase II (H-topo II) gene unexpectedly decreased (Figure 1). Examination of the time course of these events revealed that the dexamethasone-induced expression of the D-topo II gene appeared as early as 6 h after dexamethasone addition and persisted for at least 48 h (Figure 2). The associated decrease in endogenous H-topo II expression required slightly longer to detect (12 h, Figure 2b and d) but also persisted for 48 h. HBT20-parent and HBT20-MAM control cells showed no significant change in H-topo II gene expression with/without dexamethasone treatment for up to 48 h (Figure 1b, data not shown).

### *Effect of D-topo II expression on levels of D-topo II and H-topo II protein*

The results of immunoblotting analysis using antibodies to the D-topo II and H-topo II were consistent with the RNA results. In those cells that contained the D-topo II gene and were exposed to dexamethasone for 24 h, D-topo II protein was detected and the amount of immunoreactive H-topo II was decreased (Figure 3a and b).

### *DNA protein complex formation and DNA single-strand breaks produced by etoposide in HBT20-dTOP2 cells*

Indirect methods were used to assess whether D-topo II was increasing the action of topoisomerase II most closely related to etoposide cytotoxicity, that is production of topoisomerase II-DNA complexes following exposure of cells to etoposide. Small increases (20%) in etoposide-induced DNA protein cross-link formation (Table I) or etoposide-induced DNA cleavage (Table II) were seen. However, these were not significant.

### *Effect of* Drosophila *topoisomerase II gene expression on the sensitivity of HBT20 cells to etoposide and other cancer chemotherapeutic agents*

Despite the relatively minor effect of the expression of exogenous topoisomerase II on the biochemical correlates of topoisomerase II drug sensitivity, the HBT20-dTOP2 cells were significantly more sensitive to the cytotoxic actions of 10 $\mu$M etoposide following gene induction with Dex (Figure 4a). The lack of difference between the survival of transfected and non-transfected cells at the high dose of etoposide (50 $\mu$M) may simply result from the loss of assay sensitivity at high drug concentrations. Dex treatment did not alter the sensitivity of HBT20-parent or HBT20-MAM cells (Figure 4b and c). Furthermore, the transfection process did not by itself alter etoposide sensitivity since the clonogenic survival of HBT20-parent, HBT20-MAM, and HTB20-dTOP2 without Dex treatment was not statistically different (Figure 4d). Careful analysis of this effect at different times following gene induction (dexamethasone treatment) revealed that the 24 h time point was the one at which maximum sensitisation occurred (Table III). After that point in time, sensitisation diminished. This was not due to a loss of dexamethasone responsiveness by the MMTV promoter, as this persisted for at least 72 h (Figure 5). The increased sensitivity of HBT20-dTOP2 Dex-treated cells was limited to etoposide and did not extend to the topoisomerase II-reactive agent amsacrine (Table IV). This may be because of the relative insensitivity of *Drosophila* topo II to amsacrine compared with its sensitivity to etoposide (Robinson and Osheroff, 1991). The sensitivity of human and *Drosophila* topo II to etoposide is, by contrast, similar. The transfected cells also did not show an increased sensitivity to the DNA cross-linking agent cisplatin or to doxorubicin which kills cells via other mechanisms in addition to that involving topo II.

### *Cell cycle and cell growth analysis*

Cell cycle analysis showed no significant difference in HBT20-parent, HBT20-MAM and HBT20-dTOP2 cells either with or without dexamethasone treatment for 24 h (Table V). Cell growth analysis showed that the mean doubling time with standard deviation was $33 \pm 3$ h in HBT20-parent cells without Dex and $32 \pm 2$ h with Dex; $33 \pm 5$ h in HBT20-

**Figure 3** *Drosophila* (**a**) and human topoisomerase II protein (**b**) levels in HBT20-parent, HBT20-MAM and HBT20-dTOP2 cells. Cells were treated with or without dexamethasone for 24 h. *Drosophila* and human topoisomerase II protein was then assayed by immunoblot. Lane 1, HBT20-parent treated without dexamethasone for 24 h; lane 2, HBT20-parent treated with dexamethasone for 24 h; lane 3, HBT20-MAM treated without dexamethasone for 24 h; lane 4, HBT20-MAM treated with dexamethasone for 24 h; lane 5, HBT20-dTOP2 treated without dexamethasone for 24 h; lane 6, HBT20-dTOP2 treated with dexamethasone for 24 h.

**Table I** DNA–protein precipitable complex formation assessed by SDS–potassium chloride assay

| *VP-16 (μM)* | *HBT20-MAM Dex (−)* | *HBT20-MAM Dex (+)* | *HBT20-dTOP2 Dex (−)* | *HBT20-dTOP2 Dex (+)* |
|---|---|---|---|---|
| 10 | $4.0 \pm 0.2$ | $3.2 \pm 0.4$ | $4.0 \pm 0.3$ | $4.7 \pm 0.3$ |
| 50 | $6.6 \pm 0.1$ | $4.9 \pm 0.5$ | $6.5 \pm 1.1$ | $6.0 \pm 0.2$ |

Values are expressed as a ratio of [$^3$H]thymidine to [$^{14}$C]leucine in etoposide-treated cells divided by the [$^3$H] to [$^{14}$C] ratio of untreated cells with or without 24 h dexamethasone treatment. The values are the mean ± 1 s.d. from three independent experiments.

**Table II** DNA single-strand cleavage in HBT20 cells[a]

| | *Dex (−)* | *Dex (+)* |
|---|---|---|
| HBT20-parent | 253±25[b] | 268±19 |
| HBT20-MAM | 256±20 | 193±46 |
| HBT20-dTOP2 | 268±15 | 292±5 |

[a]HBT20-parent, HBT20-MAM and HBT20-dTOP2 cells were either treated with dexamethasone for 24 h or remained untreated. Then the cells were treated with 10 μM etoposide for 1 h. The DNA single-strand cleavage frequency was quantified using the alkaline elution method with proteinase (see Materials and methods). [b]rad equivalent. The values are means±s.d. from three independent experiments.

**Figure 4** Clonogenic survival in HBT20-parent, HBT20-MAM and HBT20-dTOP2 cells. Cells were pretreated with or without dexamethasone for 24 h before a 2 h etoposide exposure. The values are the mean±1 s.d. from five independent experiments.

MAM without Dex and 31±5 h with Dex; 30±2 h in HBT20-dTOP2 without Dex and 32±4 h with Dex (data from three independent experiments).

### Discussion

The present study demonstrated that transfection of the *Drosophila* topoisomerase II gene into human brain tumour cells increased the sensitivity of these cells to the cytotoxic actions of the topoisomerase II inhibitor etoposide. The *Drosophila* topoisomerase II gene was in a mammalian expression vector controlled by a dexamethasone-inducible mouse mammary tumour virus promoter (Eder *et al.*, 1993). When the pdTOP2MAMneo vector was transfected into HBT20 human brain tumour cells, expression of D-topo II mRNA and protein was demonstrated following treatment of the cells with dexamethasone (Figures 1 and 3) and sensitisation to 10 μM etoposide increased 3-fold. Thus, the increased sensitivity of the cells correlated with the induction and expression of the D-topo II gene (Table III). As previously described (Eder *et al.*, 1993), this expression

**Table III** Clonogenic survival of HBT20-MAM and HBT20-dTOP2 cells following etoposide after various durations of exposure to dexamethasone

| *Dex treatment* | *HBT20-MAM* (−) | *HBT20-MAM* (+) | *HBT20-dTOP2* (−) | *HBT20-dTOP2* (+) |
|---|---|---|---|---|
| 4 h | 0.81 ± 0.03 | 0.68 ± 0.12 | 0.77 ± 0.05 | 0.60 ± 0.19 |
| 12 h | 0.79 ± 0.11 | 0.76 ± 0.16 | 0.75 ± 0.09 | 0.43 ± 0.06* |
| 24 h | 0.51 ± 0.05 | 0.63 ± 0.14 | 0.67 ± 0.1 | 0.22 ± 0.1* |
| 48 h | 0.58 ± 0.09 | 0.71 ± 0.2 | 0.74 ± 0.16 | 0.78 ± 0.08 |
| 72 h | 0.42 ± 0.01 | 0.54 ± 0.03 | 0.48 ± 0.08 | 0.48 ± 0.01 |
| 1 week | 0.50 ± 0.01 | 0.44 ± 0.12 | 0.61 ± 0.27 | 0.72 ± 0.03 |

HBT20-MAM and HBT20-dTOP2 cells were pretreated with or without dexamethasone for 4, 12, 24, 48 and 72 h and 1 week. Dexamethasone was replaced every 48 h. After dexamethasone treatment, cells were exposed to 10 μM etoposide for 2 h. Survival fraction is calculated as stated in Materials and methods. The values are mean ± s.d. from three independent experiments. *P*-values were calculated by the Student's *t*-test. *$P < 0.05$; Dex treatment (−) *vs* Dex treatment (+).

**Figure 5** The effect of dexamethasone on transfected pMAMneo-CAT in HBT20 cells. Cells were transfected with CAT downstream from the MMTV promoter (pMAMneo-CAT) Lane 1, FBS with 10 μM dexamethasone without transfection of CAT construct; lane 2, CAT construct without MMTV promoter, with FBS and dexamethasone; lane 3, pMAMneo-CAT with FBS without 10 μM dexamethasone for 48 h; lane 4, pMAMneo-CAT with FBS and 10 μM dexamethasone for 48 h; lane 5, pMAMneo-CAT with FBS without 10 μM dexamethasone for 72 h; lane 6, pMAMneo-CAT with FBS and 10 μM dexamethasone for 72 h.

system was not absolutely conditional as small basal levels of D-topo II mRNA were detected in the absence of dexamethasone (Figure 1).

HBT20 cells were established from a brain tumour specimen from a patient who had not received prior chemotherapy. These cells were not selected for *in vitro* drug resistance, but they were relatively resistant to etoposide with an $IC_{50}$ of 13 μM following a 2 h exposure and an $IC_{50}$ of 61 μM following a 1 h exposure. Thus, we define these cells as displaying *de novo* resistance to etoposide. The aetiology of this resistance is not well understood. Herzog *et al.* (1995) have shown that altered etoposide uptake, H-topo II protein levels and H-topo II enzyme activity did not mechanistically explain the resistance. Additionally, no previously defined H-topo II mutations were identified by SSCP analysis. (M Danks, personal communication). It is therefore intriguing that transfection of a normal topo II gene onto a presumed normal topo II background altered the sensitivity of these cells. Previous investigations have been limited either to transfecting a normal gene into a mutated background to alter drug sensitivity or to transfecting the topoisomerase II gene into a cell in which the endogenous topoisomerase II gene could be inactivated by increasing the temperature (Eder *et al.*, 1993; Liu *et al.*, 1994; Wasserman and Wang, 1994).

Induction of D-topo II mRNA expression was observed as early as 6 h following stimulation with dexamethasone. This enhanced expression of D-topo II continued for as long as 48 h (Figure 2). Enhanced sensitivity to etoposide could be detected after 12 and 24 h, but not at time points greater than 24 h (Table III). Thus, at a time point when D-topo II was still being expressed (48 h), increased sensitivity to etoposide was lost.

Etoposide targets the enzyme topo II and stabilises the topo II-DNA complex. This complex is toxic to the cell. Our hypothesis was that the HBT20-dTOP2 cells were producing more topo II enzyme following dexamethasone stimulation, which resulted in more complex formation after etoposide treatment and increased cell kill. However, more complex formation was not seen in these sensitised transfected cells. This lack of concordance between etoposide-induced cytotoxicity and etoposide-induced DNA cleavage can perhaps be explained by the hypothesis recently proposed by Gerwirtz (1991). He has proposed that the site rather than the amount of drug-induced, topo II-mediated DNA cleavage dictates the cytotoxicity of any given drug treatment. It is likely that the DNA sites at which the transfected *Drosophila* enzyme act are not identical to those at which the endogenous human topoisomerase II act (Spitzner and Muller, 1988). At low concentrations of etoposide, this would mean new DNA sites would be recruited into the cytotoxic process in the *Drosophila* topoisomerase II transfected cells. Additional sites of drug action could go undetected in alkaline elution assays yet still lead to increased cytotoxicity. The higher etoposide concentration (50 μM) may be sufficiently cytotoxic so that the contribution of this small increase in sites of drug action is of little consequence.

Why then was the increase in sensitivity at the low etoposide concentration lost at 48 h, a time when gene expression was still evident and when the exogenous promoter was still functionally turned on (Figure 5)? For our hypothesis of increased cytotoxicity secondary to increased production of the target enzyme topoisomerase II to be operational, the total cellular topo II pool, not just the exogenous portion, must remain elevated. As shown in Figures 2 and 3, both the expression and the amount of endogenous human topo II protein were down-regulated following induction of the D-topo II gene. Dex treatment had no effect on the endogenous human topoisomerase II mRNA

**Table IV** Clonogenic survival of HBT20-dTOP2 cells for doxorubicin, amsacrine and cisplatin

| | *Dex treatment (−)* | *Dex treatment (+)* |
|---|---|---|
| Doxorubicin (0.1 μM) | 0.72±0.06 | 0.77±0.05 |
| Amsacrine (1.0 μM) | 0.67±0.04 | 0.65±0.07 |
| Cisplatin (1.0 μM) | 0.75±0.12 | 0.69±0.06 |
| Cisplatin (5.0 μM) | 0.47±0.01 | 0.44±0.01 |

Cells were pretreated with or without dexamethasone for 24 h, then incubated with 0.1 μM doxorubicin, 1.0 μM amsacrine or 1 or 5 μM cisplatin for 2 h. The survival fraction was calculated as described in Materials and methods. The values are mean±s.d. from two independent experiments.

**Table V** Cell cycle analysis in HBT20-parent, HBT20-MAM and HBT20-dTOP2 cells

| | *Dex* | *$G_1$ phase (%)* | *S-phase (%)* | *$G_2M$-phase (%)* |
|---|---|---|---|---|
| HBT20-parent | (−) | 67.3±6.7 | 12.5±4.2 | 20.2±5.5 |
| | (+) | 65.5±8.5 | 13.3±5.5 | 21.2±5.5 |
| HBT20-MAM | (−) | 65.9±5.0 | 12.9±5.6 | 21.2±5.8 |
| | (+) | 65.9±1.0 | 12.7±5.7 | 21.4±5.4 |
| HBT20-dTOP2 | (−) | 65.4±3.0 | 13.2±6.1 | 21.4±3.5 |
| | (+) | 72.1±6.0 | 13.5±6.0 | 14.6±5.6 |

HBT20-parent, HBT20-MAM and HBT20-dTOP2 cells were treated either with or without dexamethasone for 24 h, cell cycle analysis was then performed by flow cytometry as detailed in Materials and methods. Mean±s.d. from three independent experiments.

or protein levels in the control transfected HBT20-MAM cells (Figures 1b and 3), therefore, this down-regulation was not merely the result of Dex treatment. The observed decrease in endogenous topo II mRNA was not associated with the accumulation of cells in $G_1$ as the percentage of HBT20-dTOP2 cells in $G_1$, S and $G_2M$ was the same as seen with the HBT20-MAM and HBT20-parent cells (Table V). Dex treatment also did not alter the cell cycle time or distribution of any of the three cell lines. By 24 h of Dex stimulation, there was significantly less H-topo II protein in the HBT20-dTOP2 cells than in the HBT20-parent, the HBT20-MAM or the HBT20-dTOP2 unstimulated cells (Figure 3b). The amount of H-topo II protein detected by Western analysis following 48 h of Dex treatment was further decreased (data not shown). Therefore, while D-topo II protein may still result from transcription from the transfected gene, the amount of total cellular topo II enzyme (human + *Drosophila*) may actually be decreased. Less enzyme provides less target for etoposide interaction, resulting in less complex formation and decreased cell kill. It is tempting to speculate that the topo II gene is under tight regulatory control and that a feedback mechanism exists in cells to keep the product of this gene in balance.

In summary, we have demonstrated that transfer of a normal topo II gene into brain tumour cells having a presumed normal topo II enzyme can increase the sensitivity of these cells to etoposide. These findings have potential clinical ramifications, as they indicate that the presence of a mutated topo II enzyme in the target cell is not necessary for this manipulation to increase etoposide responsiveness. Although mutations in the topo II gene have been described in several cellular systems (Zwelling *et al.*, 1989; Bugg *et al.*, 1991; Lee *et al.*, 1992; Chan *et al.*, 1993; Champain *et al.*, 1994), no topoisomerase II mutations have been detected in specimens from patients (Kaufman *et al.*, 1994). Thus, in terms of therapeutic potential, our investigations are closely related to the clinical situation. The time frame of this etoposide sensitisation, however, was short-lived, indicating that high-efficiency gene transfer with rapid follow-up chemotherapy must be considered in any future *in vivo* application.

### Abbreviations

CAT, chloramphenicol acetyltransferase; D-topo II, *Drosophila* topoisomerase II; Dex, dexamethasone; DMEM, Dulbecco's modified Eagle medium; DMSO, dimethylsulphoxide; FCS, Fetal calf serum; HBSS, Hanks' balanced salt solution; H-topo II, human topoisomerase II; *mdr*-1, multiple drug resistance gene 1; MMTV, mouse mammary tumour virus; PBS, phosphate-buffered saline; SDS, sodium dodecyl sulphate; SSCP, single-strand conformation polymorphism; topo, topoisomerase.

### Acknowledgements

This study was supported in part by National Cancer Institute grants CA 42992 (ESK), CA 40090 (LAZ) and DHP39H (LAZ) and by Clinical Oncology Career development award 94-34 (CEH) from the American Cancer Society. We thank Drs M Blease and C Mullen for fruitful discussions and Ms Dahlia Garza for secretarial work.

### References

BLEYER WA. (1992). Principles of cancer chemotherapy in children. *Cancer Bull.*, **44,** 461–469.

BUGG BY, DANKS MK, BECK WT AND SUTTLE DP. (1991). Expression of a mutant DNA topoisomerase II in CCRF-CEM human leukemic cells selected for resistance to teniposide. *Proc. Natl Acad. Sci. USA*, **88,** 7654–7658.

CHAMPAIN JA, GOTTESMAN MM AND PASTAN I. (1994). A novel mutant topoisomerase IIα present in VP-16-resistant human melanoma cell lines has a deletion of Alanine 429. *Biochemistry*, **33,** 11327–11332.

CHAN VTW, NG S-W, EDER JP JR AND SCHNIPPER LE. (1993). Molecular cloning and identification of a point mutation in the topoisomerase II cDNA from etoposide-resistant Chinese hamster ovary cell line. *J. Biol. Chem.*, **268,** 2160–2165.

EDER JP JR, CHAN VT-W, NIEMIERKO E, TEICHER BA AND SCHNIPPER LE. (1993). Conditional expression of wild-type topoisomerase II complements a mutant enzyme in mammalian cells. *J. Biol. Chem.*, **268,** 13844–13849.

GEWIRTZ DA. (1991). Does bulk damage to DNA explain the cytostatic and cytotoxic effects of topoisomerase II inhibitors? *Biochem. Pharmacol.*, **42**, 2253–2258.

HERZOG CE, ZWELLING LA, MCWATTERS A AND KLEINERMAN ES. (1995). The expression of topoisomerase II, Bcl-2, and p53 in three human brain tumor cell lines and their possible relationship to intrinsic resistance to etoposide. *Clin. Cancer Res.*, **1**, 1391–1398.

HSIANG Y-H AND LIU LF. (1989). Evidence for the reversibility of cellular DNA lesion induced by mammalian topoisomerase II poisons. *J. Biol. Chem.*, **264**, 9713–9715.

KAUFMANN SH, MCLAUGHLIN SJ, KASTAN MB, LIU LF, KARP JE AND BURKE PJ. (1991). Topoisomerase II levels during granulocyte maturation *in vitro* and *in vivo*. *Cancer Res.*, **51**, 3534–3543.

KAUFMANN SH, KORP JE, JONER RJ, MILLER CB, SCHNEIDER E, ZWELLING LA, COWAN K, WENDEL K AND BURKE PJ. (1994). Topoisomerase II levels and drug sensitivity in adult acute myelogenous leukemia. *Blood*, **83**, 517–530.

KOHN KW, EWIG RAG, ERICKSON LC AND ZWELLING LA. (1981). Measurement of strand breaks and cross-links by alkaline elution. In *DNA Repair. A Laboratory Manual of Research Procedures*, Frieberg EC and Hanawalt PC (eds), pp.379–401. Marcel Dekker: New York.

LEE M-S, WANG JC AND BERAN M. (1992). Two independent amsacrine-resistant human myeloid leukemia cell lines share an identical point mutation in the 170kDa form of human topoisomerase II. *J. Mol. Biol.*, **223**, 837–843.

LIU LF. (1989). DNA topoisomerase poisons as antitumor drugs. *Annu. Rev. Biochem.*, **58**, 351–375.

LIU Y-X, HSIUNG Y, JANNATIPOUR M, YEH Y AND NITISS JL. (1994). Yeast topoisomerase II mutants resistant to anti-topoisomerases: identification and characterization of new yeast topoisomerase II mutants selected for resistance to etoposide. *Cancer Res.*, **54**, 2943–2951.

MINFORD J, POMMIER Y, FILIPSKI J, KOHN KW, KERRIGAN D, MATTERN M, MICHAELS S, SCHWARTZ R AND ZWELLING LA. (1986). Isolation of intercalator-dependent protein-linked DNA strand cleavage activity from cell nuclei and identification as topoisomerase II. *Biochemistry*, **25**, 9–16.

RITKE MK, ROBERTS D, ALLAN WP, RAYMOND J, BERGOLTZ VV AND YALOWICH JC. (1994). Altered stability of etoposide-induced topoisomerase II-DNA complexes in resistant human leukemia K 562 cells. *Br. J. Cancer*, **69**, 687–697.

ROBINSON MJ AND OSHEROFF N. (1991). Effects of antineoplastic drugs on the post-strand-passage DNA cleavage/religation equilibrium of topoisomerase II. *Biochemistry*, **30**, 1807–1813.

SCHNEIDER E, HORTON JK, YANG C-H, NAKAGAWA M AND COWAN KH. (1994). Multidrug resistance-associated protein gene overexpression and reduced drug sensitivity of topoisomerase II in a human breast carcinoma MCF7 cell line selected for etoposide resistance. *Cancer Res.*, **54**, 152–158.

SPITZNER JR AND MULLER MT. (1988). A consensus sequence for cleavage by vertebrate DNA topoisomerase II. *Nucleic Acid Res.*, **16**, 5533–5556.

TAKANO H, KOHNO K, ONO M, UCHIDA Y AND KUWANO M. (1991). Increased phosphorylation of DNA topoisomerase II in etoposide-resistant mutants of human cancer KB cells. *Cancer Res.*, **51**, 3951–3957.

WANG JC. (1985). DNA topoisomerases. *Annu. Rev. Biochem.*, **54**, 665–697.

WASSERMAN RA AND WANG J. (1994). Analysis of yeast DNA topoisomerase II mutants resistant to the antitumor drug amsacrine. *Cancer Res.*, **54**, 1795–1800.

WEBB CD, LATHAM MD, LOCK RB AND SULLIVAN DM. 1991). Attenuated topoisomerase II content directly correlates with a low level of drug resistance in a Chinese hamster ovary cell line. *Cancer Res.*, **51**, 6543–6549.

ZWELLING LA. (1989). Topoisomerase II as a target of antileukemia drugs: a review of controversial areas. *Hematol. Pathol.*, **3**, 101–112.

ZWELLING LA, HINDS M, CHAN D, MAYES J, SIE KL, PARKER E, SILBERMAN L, RADCLIFFE A, BERAN M AND BLICK M. (1989). Characterization of an amsacrine-resistant line of human leukemia cells: evidence for a drug-resistant form of topoisomerase II. *J. Biol. Chem.*, **264**, 16411–16420.